# Probiotic Assessment of Lactic Acid Bacteria Strains and Consortia for Enhancing Honey Bee Health and Nutrition

**DOI:** 10.3390/microorganisms14030579

**Published:** 2026-03-04

**Authors:** Gianluca Albanese, Alexandru Ioan Giurgiu, Adriana Cristina Urcan, Claudia Pașca, Tudor Nicolas Ternar, Victorița Bonta, Dalila Di Criscio, Massimo Iorizzo, Antonio De Cristofaro, Daniel Severus Dezmirean

**Affiliations:** 1 Department of Agricultural, Environmental and Food Sciences, University of Molise, Via de Sanctis snc, 86100 Campobasso, Italy; gianluca.albanese@unimol.it (G.A.); d.dicriscio@unimol.it (D.D.C.); iorizzo@unimol.it (M.I.); decrist@unimol.it (A.D.C.); 2 Department of Apiculture and Sericulture, Faculty of Animal Science and Biotechnology, University of Agricultural Sciences and Veterinary Medicine Cluj-Napoca, 400372 Cluj-Napoca, Romania; claudia.pasca@usamvcluj.ro (C.P.); tudor.ternar@usamvcluj.ro (T.N.T.); victorita.bonta@usamvcluj.ro (V.B.); ddezmirean@usamvcluj.ro (D.S.D.); 3 Department of Microbiology and Immunology, Faculty of Animal Science and Biotechnology, University of Agricultural Sciences and Veterinary Medicine Cluj-Napoca, 400372 Cluj-Napoca, Romania

**Keywords:** honey bee health, *Apis mellifera*, lactic acid bacteria, probiotic consortia, gut microbiota, apiculture, syrup supplementation

## Abstract

The decline of honey bee health has intensified interest in microbiome-based strategies to support colony resilience and reduce reliance on chemical interventions. In this study, we performed an in vitro probiotic screening of five lactic acid bacteria (LAB) strains of honey bee origin and two multi-strain consortia for prospective application in apiculture. Two formulations were evaluated: LAB Mix 1 (*Apilactobacillus kunkeei* and *Lactobacillus apis*) and LAB Mix 2 (*Lactiplantibacillus plantarum*, *Fructobacillus fructosus*, and *A. kunkeei*). Functional and safety-related traits were investigated, including auto-aggregation, cell-surface hydrophobicity, inter-strain compatibility, organic acid production, oxidative detoxification capacity, antibiotic susceptibility, haemolytic activity, and growth dynamics in sugar-based feeding syrups. All strains exhibited time-dependent increases in aggregation and hydrophobicity, with *A. kunkeei* and *F. fructosus* showing particularly strong surface-associated properties. No mutual antagonism or haemolytic activity was observed. Organic acid profiling revealed strain-specific metabolic signatures, with high lactic and citric acid production by *L. plantarum* and LAB consortia. Several strains displayed peroxidase activity, suggesting a role in oxidative stress mitigation. Growth assays demonstrated that high sugar concentrations severely limited bacterial growth, whereas moderate dilution significantly improved growth. Under osmotic stress conditions, mixed cultures generally achieved higher optical density values than individual strains. Collectively, these findings support bee-associated LAB and multi-strain formulations as promising candidates for further probiotic development.

## 1. Introduction

*Apis mellifera* L. is an insect of major ecological significance worldwide owing to its essential role as a pollinator of agricultural crops, fruit plants, and wild flora, thereby contributing to the maintenance of biodiversity [1,2]. In addition to its ecological relevance, *A. mellifera* holds substantial economic importance through its pollination services and the production of hive-derived products such as honey, wax, propolis, royal jelly, pollen, and the Romanian bee product apilarnil [3,4,5]. Over the past decade, research on the host-specific gut microbiota of bees and other pollinators has expanded considerably, leading to the identification and taxonomic characterisation of numerous bacterial species and genera [6,7,8,9]. The presence and metabolic activity of these microorganisms in the honey bee gut are associated with beneficial, probiotic-like effects, particularly among *Lactobacillus* and *Bifidobacterium* spp. The probiotic activity of these bacteria occurs through two main mechanisms: (i) enzymatic degradation of dietary carbohydrate sources, including polysaccharides, oligosaccharides, and peptides, some of which can be toxic to bees if accumulated [10,11]; and (ii) the capacity of certain microbial taxa to metabolise xenobiotic compounds, both naturally occurring and synthetic (e.g., pesticides), thereby reducing their toxicity within the gut [12]. Collectively, these processes support efficient digestion and detoxification and contribute to the overall resilience of bees, enhancing their ability to withstand nutritional and environmental stressors [13,14,15,16]. Beyond their digestive and detoxifying functions, honey bee gut bacteria confer several additional probiotic benefits, including enhanced nutrition through amino acid and vitamin synthesis [17], maintenance of intestinal homeostasis, stimulation of antimicrobial peptide expression, support of brood and honey production, and extended longevity of queens and workers [18,19,20,21]. Among these diverse effects, the most frequently reported probiotic activity involves the inhibition of both prokaryotic and eukaryotic pathogens [20,22,23,24,25,26]. The majority of bacterial strains used as probiotics belong to the lactic acid bacteria (LAB) group [27], comprising Gram-positive microorganisms characterised by strong acid tolerance and phenotypic plasticity, typically exhibiting bacillary or coccoid morphology [28]. Metabolites synthesised by LABs exert multiple beneficial effects on honey bee physiology, including modulation of immune responses and gut microbiota composition, while also displaying pronounced antimicrobial activity [19,29]. These effects are mediated through the production of organic acids (lactic, fumaric, citric, and malic), bacteriocins, hydrogen peroxide, ethanol, and diacetyl [19,30]. The antimicrobial potential of these metabolites depends not only on the producing strain but also on the physicochemical conditions established within its microenvironment [31]. A growing body of evidence has highlighted the potential of LABs as a sustainable biotechnological alternative to conventional chemical treatments for the prevention and management of honey bee diseases. Such approaches represent promising and environmentally sound strategies for improving colony health and resilience.

This study aimed to evaluate the probiotic potential of two formulations comprising lactic acid bacteria, *Apilactobacillus kunkeei* and *Lactobacillus apis* (LAB mix 1); and *Lactiplantibacillus plantarum*, *Fructobacillus fructosus*, and *A. kunkeei* (LAB mix 2), systematically comparing single strains and multi-strain formulations under sugar-feeding conditions relevant to apicultural practice. Specifically, we examined interstrain biocompatibility and the ability of the selected isolates to withstand high-sugar environments, mimicking conditions typical of supplementary bee diets. Furthermore, we characterised key functional and safety-related attributes, including auto-aggregation, cell-surface hydrophobicity, antibiotic susceptibility, hydrogen peroxide and organic acid production, hemolytic activity, and mutual antagonistic interactions. These evaluations collectively enabled the assessment of the suitability and functional robustness of the selected LAB strains as promising probiotic candidates for apicultural applications.

## 2. Materials and Methods

### 2.1. Bacterial Strains

Five lactic acid bacteria (LAB) strains were used in this study, including three isolates derived from the gut microbiota of the Western honeybee (*Apis mellifera*) and two reference type strains. The honeybee gut isolates are preserved in the culture collection of the Di.A.A.A. (Department of Agricultural, Environmental and Food Sciences, University of Molise, Italy). These strains are *Lp. plantarum* A1H1B2, *A. kunkeei* ST56, and *F. fructosus* 346, with accession numbers PP400696, PP379895, and PP379896, respectively. The two reference strains were previously characterised within the PN-III-P1-1.1-PD-2019-0498 project (Contract No. 140/2020) at the Department of Apiculture and Sericulture and are currently maintained in the culture collection of the Faculty of Animal Science and Biotechnology, at the University of Agricultural Sciences and Veterinary Medicine of Cluj-Napoca (Romania). These Romanian reference strains are *A. kunkeei* ATCC 700308 (hereafter *A. kunkeei* C1) and *L. apis* DSM 26264 (hereafter *L. apis* C2). Two LAB consortia were formulated for the experiments. LAB Mix 1 consisted of *A. kunkeei* C1 and *L. apis* C2, whereas LAB Mix 2 comprised *L. plantarum* A1H1B2, *A. kunkeei* ST56, and *F. fructosus* 346. In both cases, the LAB mixes were prepared by combining equal volumes of broth cultures adjusted to a final concentration of 1.5 × 10^8^ CFU/mL (0.5 McFarland).

### 2.2. Cell Surface Properties

#### 2.2.1. Culture Preparation and Standardisation

LAB strains were cultivated in de Man, Rogosa, and Sharpe (MRS) broth at 35 °C for 12 h to obtain log-phase cultures. Following incubation, cells were harvested by centrifugation at 8000 rpm for 15 min at 4 °C, washed three times with sterile physiological saline (0.9% NaCl), and resuspended in the same solution to eliminate residual medium components and metabolites. The bacterial suspensions were then adjusted to an optical density (OD) of 0.5 at 580 nm, corresponding to approximately 1 × 10^8^ CFU/mL, as calibrated using the McFarland turbidity standard. Optical density measurements were obtained with a spectrophotometer (Multilabel Counter, model 1420; PerkinElmer, San Jose, CA, USA). LAB Mix 1 and LAB Mix 2 were prepared by combining the respective LAB strains to a final concentration of 1 × 10^8^ CFU/mL per bacterial component.

#### 2.2.2. Auto-Aggregation Assay

The auto-aggregation (AA) ability of LAB strains was evaluated following Cozzolino et al. [32], with minor modifications. Standardised bacterial suspensions were incubated at 35 °C, and the optical density at 580 nm (OD_580_) was recorded after 1, 2, 5, and 24 h using a spectrophotometer (Multilabel Counter, model 1420; PerkinElmer, San Jose, CA, USA). Auto-aggregation was expressed as a percentage (AA%) calculated according to the equation:

AA% = [1 − (OD_t_/OD_0_)] × 100

where OD_0_ is the initial absorbance and OD_t_ the absorbance at each time point (1–24 h). Higher AA% values indicate greater self-aggregation capacity, reflecting potential for colonisation and persistence in the host gut environment.

#### 2.2.3. Hydrophobicity

Cell surface hydrophobicity was assessed using the bacterial adhesion to hydrocarbons (BATH) method described by Iorizzo et al. [33] employing xylene and toluene as solvents. Equal volumes (1:1, *v*/*v*) of bacterial suspension and hydrocarbon were vortexed vigorously for 5 min to promote phase interaction. The mixtures were allowed to separate at room temperature, and the aqueous phase was carefully collected after 15, 30, and 60 min. Absorbance of the aqueous phase was measured at 580 nm using a spectrophotometer (Multilabel Counter, model 1420; PerkinElmer, San Jose, CA, USA). Hydrophobicity (H%) was calculated as:

H% = [1 − (OD_t_/OD_0_)] × 100

where OD_0_ represents the absorbance before solvent addition and OD_t_ the absorbance after each incubation time. Elevated H% values denote a more hydrophobic bacterial surface and stronger affinity for hydrocarbon phases, traits commonly associated with enhanced adherence to host epithelial tissues.

### 2.3. Functional and Safety Characterisation of LAB Strains

#### 2.3.1. Interstrain Antagonistic Interactions

Mutual antagonistic activity among LAB strains was evaluated according to the method of Urcan et al. [34]. LAB cultures were standardised to a turbidity corresponding to a 0.5 McFarland standard (≈1.5 × 10^8^ CFU/mL) and uniformly spread on MRS agar plates (Sigma-Aldrich, Darmstadt, Germany) using sterile disposable spreaders. Plates were incubated at 35 °C for 48 h to allow colony formation. Subsequently, 6 mm agar discs were aseptically excised and placed onto fresh MRS agar plates previously inoculated with different LAB strains. After incubation under identical conditions, the inhibition zones surrounding each disc were measured (mm). Antagonistic activity was categorised as follows: strong (+++, >11 mm), moderate (++, 6–10 mm), weak (+, 1–5.9 mm), or absent (−, 0 mm). All experiments were performed in triplicate.

#### 2.3.2. Hemolysis Assay

The hemolytic potential of LAB strains was determined following the method described by Yasmin et al. [35], with minor adaptations. LAB cultures were streaked onto MRS agar plates supplemented with 5% defibrinated horse blood, prepared both with and without fructose, and incubated at 37 °C for 24–48 h. *Staphylococcus aureus* ATCC 25923 served as a positive control. Hemolysis was classified based on the appearance of the colonies and surrounding zones: α-hemolysis (greenish discoloration), β-hemolysis (complete clearing), and γ-hemolysis (no visible change). Each assay was conducted in duplicate. The absence of β-hemolysis was considered indicative of probiotic safety.

#### 2.3.3. Assessment of Catalase and Peroxidase Activities in LAB Isolates

Catalase and peroxidase activities were determined using the Catalase/Oxy Test (Liofilchem^®^, Roseto degli Abruzzi, Italy) [27,36], following the manufacturer’s instructions. Briefly, 0.1 g of desiccated chlorpromazine reagent was rehydrated with four drops of 0.85% saline solution at room temperature. Freshly grown LAB colonies (18–24 h) were collected with sterile loops and suspended in the rehydrated reagent. Three drops of the supplied hydrogen peroxide (H_2_O_2_) solution were added. The immediate formation of gas bubbles or foam indicated catalase activity, while a pink to red colour change within one minute denoted peroxidase activity. All assays were performed in duplicate under aseptic conditions and results were interpreted visually.

#### 2.3.4. Antibiotic Resistance Profile of LAB

The antibiotic susceptibility of LAB isolates was assessed using the Kirby–Bauer disk diffusion method on MRS agar (Sigma-Aldrich, Darmstadt, Germany), supplemented with fructose when required to support strain growth. Commercial antibiotic disks were obtained from Oxoid (Thermo Fisher Scientific, Basingstoke, UK) and used according to the manufacturer’s specifications. Disk concentrations were selected in accordance with Clinical and Laboratory Standards Institute (CLSI) and European Committee on Antimicrobial Susceptibility Testing (EUCAST) recommendations, as well as previously published protocols for phenotypic antibiotic susceptibility testing of lactic acid bacteria. The antibiotics tested included: ampicillin (10 µg), chloramphenicol (30 µg, 50 µg), erythromycin (10 µg), enrofloxacin (10 µg), gentamicin (30 µg), kanamycin (30 µg), nalidixic acid (concentration as per reference standards), oxytetracycline (30 µg), streptomycin (10 µg, 100 µg), sulfathiazole (30 µg), tetracycline (30 µg), and vancomycin (30 µg).

Overnight LAB cultures were adjusted to a 6.0 McFarland standard (≈1 × 10^9^ CFU/mL) and spread evenly on the agar surface using sterile spreaders. Plates were incubated at 35 °C for 24 h, and inhibition zones were measured (mm). Interpretation was based on previously established criteria, with isolates classified as susceptible (S), moderately susceptible (MS), or resistant (R). All assays were conducted in triplicate to ensure reproducibility.

#### 2.3.5. Viability of LABs in Concentrated Sugar Solutions

To evaluate the suitability of sugar-based bee feeding syrups as delivery matrices for presumptive probiotic LABs, bacterial growth was assessed in two carbohydrate-rich substrates: a laboratory-prepared sucrose syrup (SS) and a commercial beekeeping syrup (ApiSyrup; SC APIS SRL, Blaj, Romania). The sucrose syrup was prepared by dissolving food-grade sucrose (Sigma-Aldrich, Darmstadt, Germany) in sterile distilled water at a ratio of 2:1 (*w*/*v*), corresponding to concentrations commonly used in apicultural feeding practices. ApiSyrup (AS) consisted of 72.4% dry matter (glucose 53.6%, fructose 42.9%), according to the manufacturer’s specifications. All syrup preparations were sterilized by filtration through 0.22 µm membrane filters (Merck Millipore, Burlington, MA, USA) to avoid thermal degradation. Each syrup was tested at three concentrations: undiluted, 1:1 (*v*/*v*) dilution with sterile distilled water, and 1:2 (*v*/*v*) dilution, in order to simulate different osmotic conditions relevant to practical beekeeping applications. LAB cultures grown overnight in MRS broth were harvested, washed twice in sterile physiological saline (0.9% NaCl), and inoculated into the test matrices at an initial concentration of approximately 1 × 10^8^ CFU/mL. Mixed cultures (LAB Mix 1 and LAB Mix 2) were prepared by combining individual strains in equal proportions prior to inoculation.

All assays were conducted in sterile 96-well microplates and incubated at 35 °C for 48 h under static conditions. Bacterial growth was monitored by measuring optical density at 600 nm (OD_600_) at 15 min intervals using a Multilabel Counter spectrophotometer (PerkinElmer, San Jose, CA, USA). MRS broth inoculated with the corresponding LAB served as positive growth controls, while uninoculated syrups and MRS medium served as negative controls. Optical density measurements were used to assess relative biomass increase and growth kinetics under different syrup concentrations. This approach provides a comparative assessment of osmotic tolerance among strains and formulations. It should be noted that OD_600_ values provide an indirect estimate of bacterial growth and do not directly quantify viable cell numbers.

#### 2.3.6. Profiling of Lactic Acid Bacteria-Derived Organic Acids via HPLC

The organic acid profiles produced by the bacterial strains investigated in this study were determined by analysing the cell-free supernatant (CFS) using HPLC. Briefly, bacterial cultures were inoculated at 1% (*v*/*v*) into MRS broth supplemented with fructose and incubated for 24 h at 35 °C. Following incubation, samples were centrifuged at 10,000 RPM for 15 min, and the supernatants were subsequently filtered through sterile 0.22 µm syringe filters to obtain the CFS. Quantification of organic acids was performed using a SHIMADZU VP series HPLC system equipped with a photodiode array (PDA) UV-Vis detector (Shimadzu Instruments, Kyoto, Japan). Separation was achieved on a Waters Atlantis dC18 column (250 mm × 4.6 mm, 5 µm particle size) under isocratic conditions. The mobile phase consisted of 15 mM phosphate buffer (pH 2.4), adjusted with concentrated sulfuric acid. The flow rate was 0.6 mL/min, the injection volume was 10 μL, and detection was carried out at 202 nm. Column temperature was maintained at 30 °C throughout the analysis. The organic acids analysed included lactic, acetic, citric, succinic, and propionic acids. Identification was based on retention times and UV spectra compared to authentic standards. Quantification was performed using individual calibration curves prepared for each acid. The retention time of each reference standard, relative standard deviation in retention time (RSD %), R^2^, LOD, and LOQ are reported in Table 1. All results are expressed as grams per litre (g/L).

#### 2.3.7. Statistical Analysis

Statistical analyses were conducted using RStudio (R version 4.5.0; R Core Team 2023 [37]). Data preprocessing and visualisation were performed with the tidyverse suite, and inferential statistics were carried out using rstatix, with graphical annotation via ggpubr. All data from three independent experiments are presented as mean ± standard deviation (SD). Growth kinetics were evaluated using one-sample, one-sided *t*-tests on normalised OD_600_ values at each time point, with Bonferroni correction applied for multiple testing. Given the exploratory nature of the analysis and predefined directional hypothesis (growth reduction under osmotic stress), one-sided testing was considered appropriate. Hydrophobicity and auto-aggregation datasets, confirmed to be normally distributed, were analysed using two-way ANOVA to assess the effects of strain, time, and their interaction, followed by Tukey’s post hoc test. Statistical significance was defined as *p* < 0.05.

## 3. Results and Discussions

### 3.1. Cell-Surface Properties

**Figure 1:**
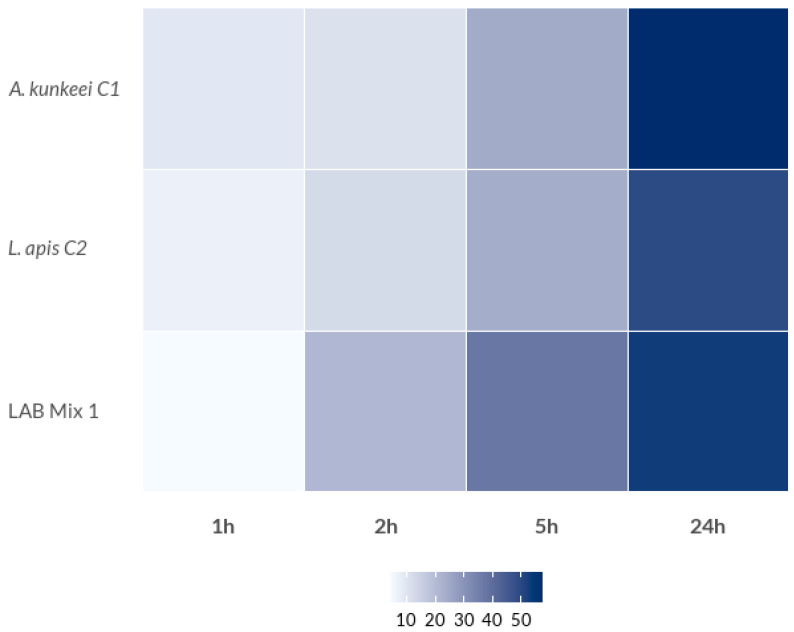
Percentage of auto-aggregation (AA%) of LAB strains (*A. kunkeei* C1 and *L. apis* C2) and LAB Mix 1.

**Figure 2:**
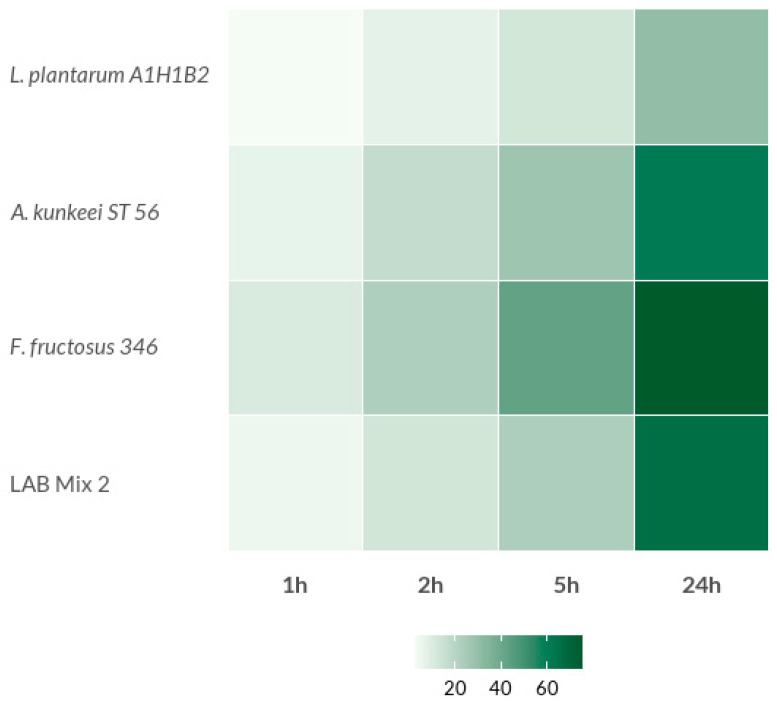
Percentage of auto-aggregation (AA%) of the LAB strains (*L. plantarum* A1H1B2, *A. kunkeei* ST 56 and *F. fructosus* 346) and LAB Mix 2.

**Figure 3:**
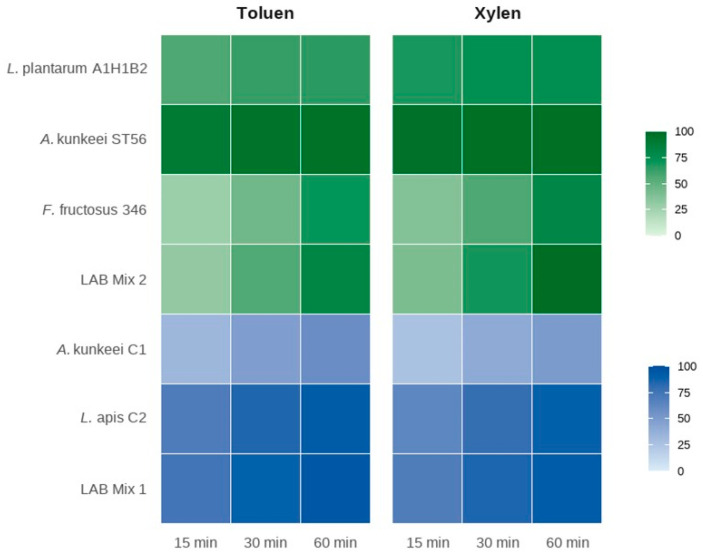
Adhesion of the LAB strains to toluene and xylene expressed as hydrophobicity (%) after different contact times (15, 30, and 60 min).

The auto-aggregation capacity and surface hydrophobicity of the tested LAB strains demonstrated a consistent, time-dependent increase across all isolates and experimental groups, though the extent of aggregation varied significantly among strains (Figure 1, Figure 2 and Figure 3; Supplementary Tables S1–S4).

These parameters are commonly employed as preliminary in vitro descriptors of cell-surface properties in probiotic screening, as they are associated with bacterial adhesion, biofilm formation, and persistence within host environments, including the honeybee gut [9]. In the first dataset, comprising *A. kunkeei* C1, *L. apis* C2, and the mixed culture LAB Mix 1, auto-aggregation values were initially low (4.0–8.7% at 1 h) but increased markedly over time. After 24 h, *A. kunkeei* C1 displayed the highest auto-aggregation (57.9%), followed by LAB Mix 1 (54.0%) and *L. apis* C2 (49.3%) (Figure 1; Supplementary Table S1).

Similarly, in the second group, which included *Lp. plantarum* A1H1B2, *A. kunkeei* ST56, *F. fructosus* 346, and LAB Mix 2, auto-aggregation increased progressively over time, ranging from 2.2–10.2% at 1 h to substantially higher values after 24 h (Figure 2; Supplementary Table S2). Notably, both *A. kunkeei* strains exhibited strong self-aggregation behavior, suggesting that this species may possess highly adhesive surface-associated features, such as specific cell surface proteins or exopolysaccharides, that facilitate cell–cell interactions. These findings are consistent with previous reports describing the aggregation ability of bee-associated LAB and highlighting its importance for probiotic adhesion and colonisation potential [38,39,40], although the extrapolation of these in vitro traits to gut colonisation should be interpreted with caution and warrants confirmation using honey bee-relevant adhesion models. The pronounced auto-aggregation observed for *A. kunkeei* and *F. fructosus* may reflect their ecological adaptation to the sugar-rich environments of the hive and the honeybee gut, where aggregation and biofilm formation can confer selective advantages. The intermediate aggregation levels observed in the mixed cultures likely indicate functional compatibility among the strains rather than the presence of strong synergistic effects.

The high aggregation capacity observed for these strains is consistent with their elevated surface hydrophobicity, supporting a functional relationship between these two surface-associated properties. Hydrophobicity assays performed with toluene and xylene confirmed the aggregation results and highlighted solvent- and strain-dependent behaviours (Figure 3).

In the first dataset, LAB Mix 1 and *L. apis* C2 exhibited the highest affinity for both solvents, with values of 94.5% and 91.8% for toluene and 92.0% and 88.2% for xylene, respectively, whereas *A. kunkeei* C1 showed moderate hydrophobicity (~58.9%). In the second dataset, *A. kunkeei* ST56 and LAB Mix 2 displayed exceptionally high hydrophobicity, exceeding 90% with both solvents and reaching up to 99.1% with xylene after 60 min. In contrast, *F. fructosus* 346 and *Lp. plantarum* A1H1B2 demonstrated moderate but progressively increasing hydrophobicity over time. Overall, these findings are consistent with the well-established correlation between high surface hydrophobicity and enhanced aggregation and in vitro adhesion potential in probiotic LABs [41,42,43].

From a functional standpoint, high auto-aggregation and hydrophobicity are advantageous properties for LAB intended as probiotics in the honeybee diet. These traits may support bacterial adherence to the crop or gut epithelium, facilitate colonisation, biofilm formation, and competitive exclusion of pathogens, all desirable for dietary supplementation in bees [19,44,45]. However, in the absence of honey bee-relevant adhesion models or in vivo persistence assessments, the current findings do not establish gut colonization and should be interpreted strictly as indicative of surface-associated traits observed in vitro. The robust aggregation and hydrophobic profiles observed in *A. kunkeei* and *F. fructosus* mark them as particularly promising candidates, and the mixed cultures also displayed intermediate-to-high performance, indicating possible synergistic interactions among combined strains. These combined observations suggest that both single and mixed LAB cultures possess favourable surface properties, although their intensity and kinetics differ according to strain composition and solvent interaction.

### 3.2. Mutual Antagonistic Activity

This assay was conducted to determine whether the LAB strains included in this study were suitable for use in combination as a potential biopreparative formulation. None of the tested strains exhibited antagonistic activity against one another, as no inhibition zones were observed. This finding is particularly relevant, as it indicates that the strains can coexist without competitive suppression and may therefore act synergistically. Furthermore, the absence of mutual inhibition suggests that these LAB do not produce metabolites that impair each other’s growth, supporting their suitability for inclusion in a single probiotic preparation intended for apicultural applications.

### 3.3. Organic Acid Profile

**Figure 4:**
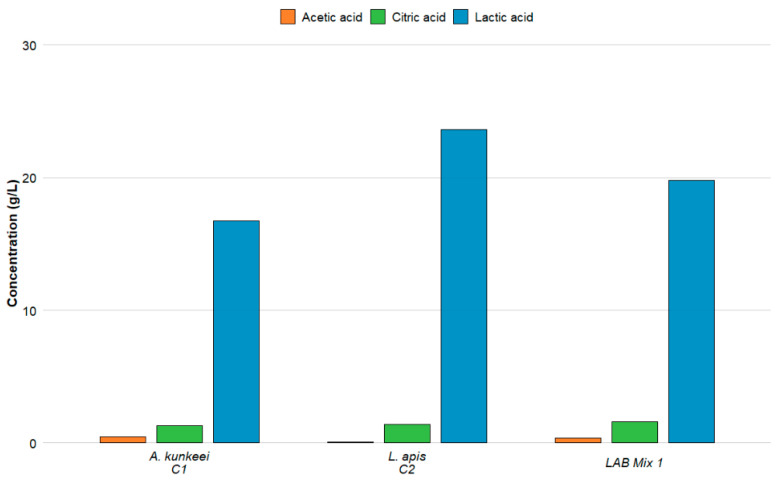
Organic acid profiles of the strains C1, C2 and LAB Mix 1.

**Figure 5:**
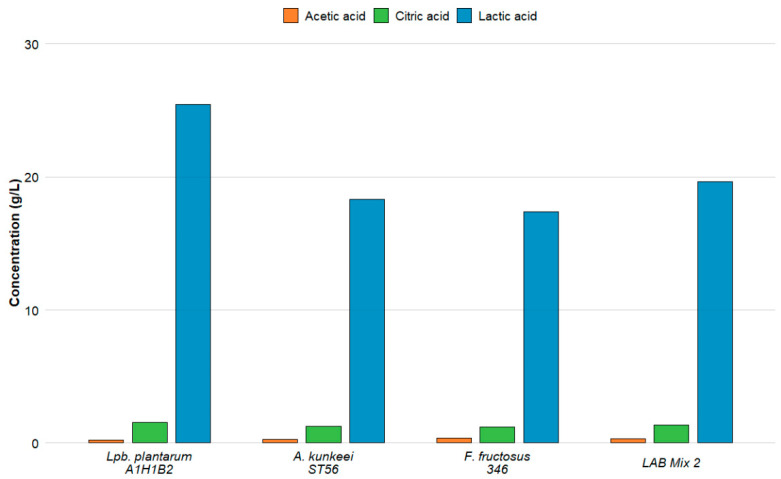
Organic acid profiles of the strains A1H1B2, ST56, 346 and LAB Mix 2.

In this study, we characterised the *de novo* organic acid profiles of five LAB strains and two mixed cultures (Figure 4 and Figure 5; Supplementary Table S5). Culture supernatant pH was not measured.

Lactic, acetic, and citric acids were detected in all tested samples, while other organic acids were below the detection limit under the applied experimental conditions. The strains displayed clear and statistically significant differences in their metabolic output (*p* < 0.05). *Lp. plantarum* A1H1B2 produced the highest lactic acid concentration (25.46 g/L), followed by *L. apis* C2 (23.61 g/L) and the two LAB mixtures (~19.8 g/L), whereas strains belonging to *A. kunkeei* and *F. fructosus* generated substantially lower amounts. Citric acid production exhibited a comparable pattern: *Lp. plantarum* A1H1B2 and LAB Mix 1 yielded the highest concentrations (1.57–1.58 g/L), while *F. fructosus* 346 and *A. kunkeei* ST56 produced the lowest. Acetic acid output varied the most across strains, with *A. kunkeei* C1 producing the highest concentration (0.44 g/L) and *L. apis* C2 the lowest (0.08 g/L), both significantly distinct from all other isolates. These strain-specific metabolic signatures carry important implications for honey bee health. Organic acids, particularly lactic and acetic acids, have been reported, in previous studies, to contribute to the inhibition of *Paenibacillus larvae*, with antimicrobial effects largely attributable to pH-dependent mechanisms, as suggested by the loss of inhibitory activity following CFS neutralisation [46,47]. Furthermore, according to previously published literature, organic acids and LAB supplementation have been associated with the modulation of microsporidian infections such as *Nosema ceranae* [19,48,49]; however, such pathogen-specific effects were not experimentally evaluated in the present study. In support of this previously reported evidence, supplementation with LAB strains has been reported to reduce *N. ceranae* levels in honey bees under laboratory conditions, and commensal bacteria such as *A. kunkeei* have been used in in-hive trials to reduce nosemosis [21]. Furthermore, the ecological significance of LAB in the honey bee gut during *Nosema* infection is supported by findings that *N. ceranae* disturbs the native gut microbiota, decreasing the abundance of key lactic acid bacteria (such as *F. fructosus* and *Lactobacillus* spp.) in infected bees [19,50].

The presence of citric acid, in addition to lactic and acetic acids, may contribute to acid-mediated mechanisms that have been linked to pathogen suppression in the literature, potentially enhancing acid stress or supporting the production of other antimicrobial compounds by LAB. Thus, strains producing high levels of these acids, such as *Lp. plantarum* A1H1B2 and LAB mixtures, may be considered promising candidates within a preliminary probiotic screening framework and require further validation in pathogen-targeted assays before any biocontrol-related conclusions can be drawn. Overall, these findings are consistent with the concept that LAB-derived organic acids are associated with mechanisms linked to pathogen suppression reported in the literature, supporting the development of microbiome-based strategies aimed at improving honey bee health and colony resilience.

### 3.4. Oxidative Detoxification Potential of the Tested LAB

The catalase- and peroxidase-activity assay revealed that among the five LAB strains, only a subset exhibited enzymatic activity. Specifically, strains *Lp. plantarum* A1H1B2, *A. kunkeei* ST56, *A. kunkeei* C1, and *L. apis* C2 displayed positive peroxidase activity, while none of the strains showed catalase activity under the tested conditions. *F. fructosus* 346 was negative in both assays. These results suggest that while these LABs do not degrade hydrogen peroxide via classical catalase, several are capable of peroxidase-mediated hydrogen peroxide detoxification. This enzymatic profile holds significant implications for probiotic functionality and oxidative stress tolerance. It is well-known that many lactic acid bacteria are catalase-negative under standard conditions, as catalase activity in LAB often depends on the presence of heme or other cofactors [51]. Some LAB species, however, can express peroxidase or pseudo-catalase activities to neutralise hydrogen peroxide and mitigate oxidative stress. In food-derived LAB, peroxidase and manganese-dependent catalase activities have been isolated and characterised, supporting their role in H_2_O_2_ scavenging (e.g., *Pediococcus pentosaceus* displays NADH peroxidase and Mn-catalase activity) [52]. From an apicultural probiotic perspective, peroxidase-positive LAB may contribute to the oxidative resilience of the formulation. In the honey bee gut environment, reactive oxygen species, including hydrogen peroxide, can originate both from host metabolism and from other microbial activities. LAB capable of peroxidase-mediated detoxification may thereby help maintain redox homeostasis, potentially enhancing their survival and persistence [19,34]. Importantly, the absence of catalase does not necessarily detract from the utility of these strains: as they still possess a mechanism to mitigate hydrogen peroxide via peroxidase, they may remain viable and functional within a complex microbial ecosystem. Indeed, other studies in apiculture have documented the use of catalase- and peroxidase-negative, but metabolically robust, LAB strains with probiotic potential [34,53].

The enzymatic phenotype characterised here, peroxidase-positive and catalase-negative, in most strains, suggests these LAB are likely to mitigate oxidative stress through peroxidase-dependent pathways. This trait may enhance their suitability for inclusion in probiotic consortia aimed at improving honeybee health, particularly under oxidative or stress-inducing conditions.

### 3.5. Hemolytic Activity of LAB Strains

The hemolysis assay demonstrated that all tested LAB strains exhibited γ-hemolysis (no visible hemolytic zones), indicating the absence of hemolytic activity, whereas the positive control (*S. aureus* ATCC 25923) showed clear β-hemolysis. This result strongly supports the safety profile of the strains, as non-hemolytic behaviour is widely regarded as a key criterion in selecting probiotic LAB [4,54]. From a biotechnological standpoint, the γ-hemolytic phenotype indicates that these LAB are unlikely to produce cytolysins or other virulence-associated hemolysins [55], and therefore can be safely considered for formulation in probiotic preparations. In particular, for apicultural applications, using non-hemolytic LAB is essential to avoid any risk of host–microbe cytotoxicity, making these strains appropriate candidates for further development. Importantly, this result is consistent with the behavior of LAB naturally associated with honey bees: for example, *A. kunkeei* strains isolated from *Apis cerana* bee bread were found to be non-hemolytic and devoid of virulence factors in vitro [56]. In another study, LAB strains isolated from *Apis mellifera* subsp. *intermissa* (including *A. kunkeei* and *F. fructosus*) showed no hemolytic activity, further supporting their safety for probiotic use [38]. Taken together, these findings confirm the absence of hemolytic activity among the investigated LAB strains, supporting their in vitro safety profile.

### 3.6. Susceptibility to Antibiotics

**Figure 6:**
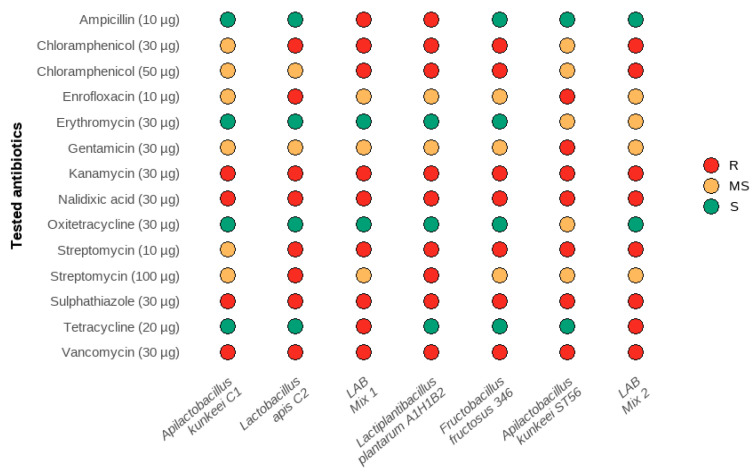
Antibiotic susceptibility of the five LAB strains, and their mixes (LAB Mix 1–2 evaluated by Kirby–Bauer disk diffusion assay (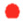 resistant: R; 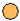 medium sensitive: MS; 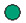 sensitive: S).

The susceptibility of five LAB strains, and their mixes (LAB Mix 1–2), to 12 antibiotics was evaluated using the Kirby–Bauer disk diffusion method (Figure 6). Phenotypic responses were interpreted descriptively to assess resistance patterns relevant to preliminary probiotic safety evaluation. The efficacy of each antibiotic depended on both the tested antibiotic and its concentration. Distinct susceptibility profiles were observed among individual strains and between the two multi-strain formulations. Overall, *A. kunkeei* C1 and *L. apis* C2, which together form LAB Mix 1, displayed a largely favourable profile, showing sensitivity to tetracyclines, ampicillin and erythromycin with only moderate responses to some aminoglycosides and phenicols. In contrast, the strains included in LAB Mix 2 (*Lp. plantarum* A1H1B2, *F. fructosus* 346 and *A. kunkeei* ST56) produced a more heterogeneous pattern with a broader spectrum of resistance (notably to vancomycin, kanamycin and nalidixic acid) that reflects strain-dependent variability.

These observations are consistent with genus-level surveys showing that many LABs exhibit intrinsic resistance to glycopeptides (vancomycin) and aminoglycosides, and that susceptibility to tetracyclines, macrolides and phenicols is strain-specific rather than species-uniform [57]. Strain-level data may inform consideration for probiotic application in apiculture: *A. kunkeei* strains have been documented as bee symbionts with variable but generally acceptable susceptibility profiles and additional beneficial traits (e.g., production of bacteriocins), suggesting potential probiotic relevance provided that careful safety screening is performed [58,59]. *F. fructosus* isolates, fructophilic LAB common in honey and hive environments, tend to show intrinsic resistance patterns similar to other bee-derived LAB but remain sensitive to several clinically relevant antibiotics in many cases, a combination that may support their evaluation for dietary supplementation while highlighting the need to verify the absence of mobile resistance determinants [38,40]. Similarly, *Lp. plantarum* isolates frequently exhibit intrinsic vancomycin resistance linked to peptidoglycan chemistry (D-Ala–D-Lac/Ala differences) and show variable resistance to tetracyclines or chloramphenicol depending on strain and provenance; thus, individual strain phenotyping, rather than species-level assumptions, is essential prior to any application [57,60]. From an applied perspective, the predominance of intrinsic, non-transferable resistance phenotypes in many LAB is reassuring in the context of probiotic safety; however, the documented capacity of some LABs to carry transferable *tet*/*erm* genes in particular contexts mandates genomic screening for mobile resistance elements prior to field use [57,61]. Finally, because antibiotic treatments (e.g., oxytetracycline) can perturb honeybee gut communities and host fitness, probiotic strains that are phenotypically stable and lack mobilisable resistance genes could help restore microbiome balance following exposure. However, any candidate strain must undergo both phenotypic susceptibility testing (as performed here) and molecular screening for resistance determinants before recommendation for supplementation in the honeybee diet [61,62]. Antibiotic susceptibility profiling in the present study was conducted exclusively using phenotypic disk diffusion assays, which provide an initial screening of resistance patterns but do not allow discrimination between intrinsic and potentially transferable resistance mechanisms. Although this approach is commonly used in preliminary characterisation of lactic acid bacteria, it does not replace minimum inhibitory concentration (MIC) testing or molecular screening for known transferable resistance determinants, such as *tet* or *erm* genes. Consequently, the safety assessment presented here should be considered preliminary, and further investigations based on whole-genome sequencing and targeted resistance gene analysis are required.

### 3.7. Suitability of Sugar-Based Bee Feeding Syrups for LAB Probiotic Delivery

The growth kinetics of individual LAB strains (ST56, A1H1B2, 346, C1, C2) and mixed cultures (LAB Mix 1 and LAB Mix 2) were evaluated in two carbohydrate-rich matrices: laboratory-prepared sucrose syrup (SS; sucrose:water 2:1, 1:1, 1:2) and a commercial beekeeping formulation (ApiSyrup; AS) at three concentrations (undiluted, 1:1, 1:2). Growth was monitored for 48 h by OD_600_ measurements at 15 min intervals. The pH of the sugar solutions was not monitored during the incubation period.

**Figure 7:**
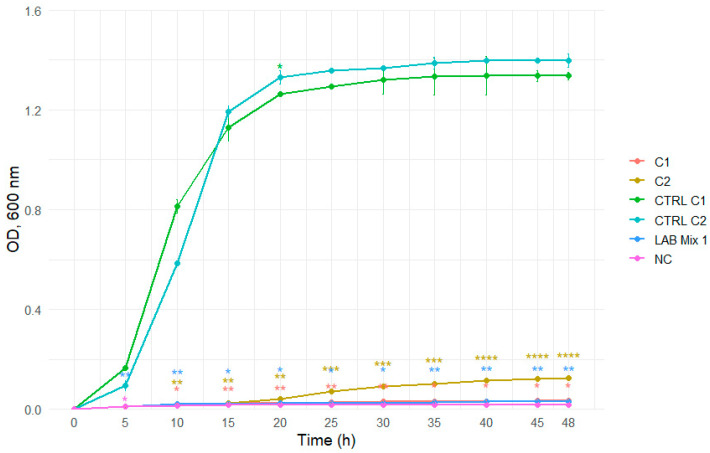
Growth curves of lactic acid bacteria strains C1, C2, and the mixed culture LAB Mix 1 in sucrose syrup at dilutions 2:1. Growth kinetics were recorded over 48 h and represent the mean of four biological replicates. Asterisks indicate statistical significance versus T0 (one-sample *t*-test, Bonferroni corrected). Significance levels are coded as follows: * *p* < 0.05; ** *p* < 0.01; *** *p* < 0.001; **** *p* < 0.0001.

**Figure 8:**
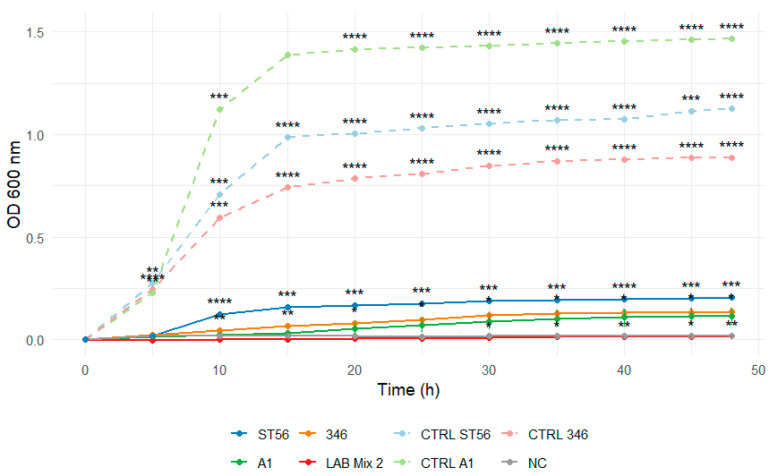
Growth curves of lactic acid bacteria strains A1, 346, ST56, and the mixed culture LAB Mix 2 in sucrose syrup at dilutions 2:1. Growth kinetics were recorded over 48 h and represent the mean of four biological replicates. Asterisks indicate statistical significance versus T0 (one-sample *t*-test, Bonferroni corrected). Significance levels are coded as follows: * *p* < 0.05; ** *p* < 0.01; *** *p* < 0.001; **** *p* < 0.0001.

In 2:1 SS, all strains, including mixed cultures, displayed extended lag phases and minimal increases in optical density throughout the incubation period, reflecting strong osmotic inhibition (Figure 7 and Figure 8).

**Figure 9:**
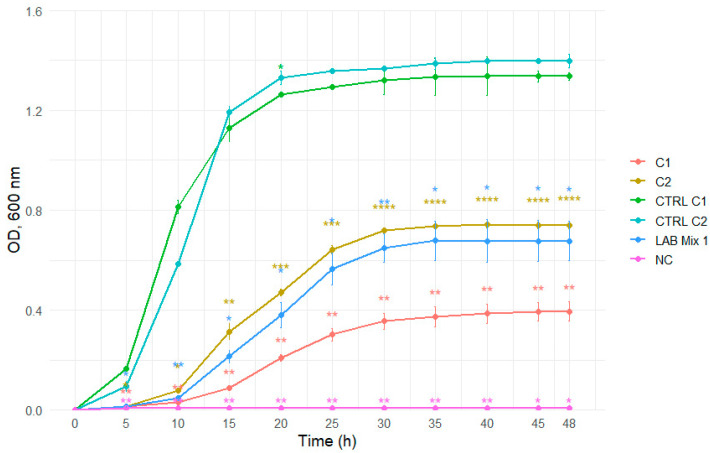
Growth curves of lactic acid bacteria strains C1, C2, and the mixed culture LAB Mix 1 in sucrose syrup at dilutions 1:1. Growth kinetics were recorded over 48 h and represent the mean of four biological replicates. Asterisks indicate statistical significance versus T0 (one-sample *t*-test, Bonferroni corrected). Significance levels are coded as follows: * *p* < 0.05; ** *p* < 0.01; *** *p* < 0.001; **** *p* < 0.0001.

**Figure 10:**
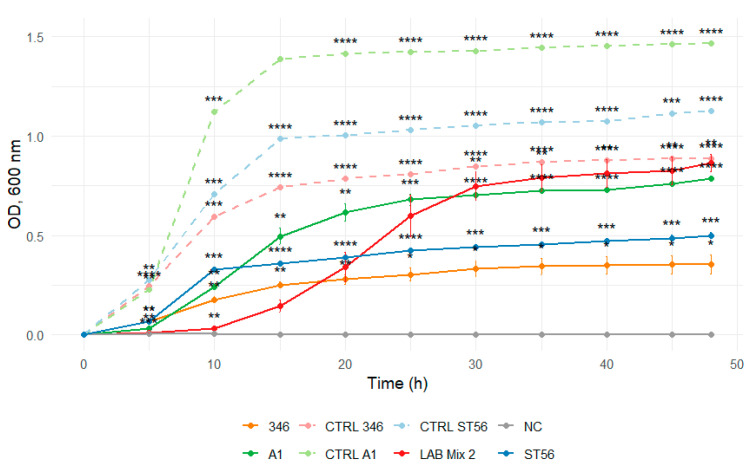
Growth curves of lactic acid bacteria strains A1, 346, ST56, and the mixed culture LAB Mix 2 in sucrose syrup at 1:1 dilution. Growth kinetics were recorded over 48 h and represent the mean of four biological replicates. Asterisks indicate statistical significance versus T0 (one-sample *t*-test, Bonferroni corrected). Significance levels are coded as follows: * *p* < 0.05; ** *p* < 0.01; *** *p* < 0.001; **** *p* < 0.0001.

Dilution to 1:1 SS reduced the inhibitory effect, evidenced by shorter lag phases and markedly enhanced growth across strains (Figure 9 and Figure 10).

**Figure 11:**
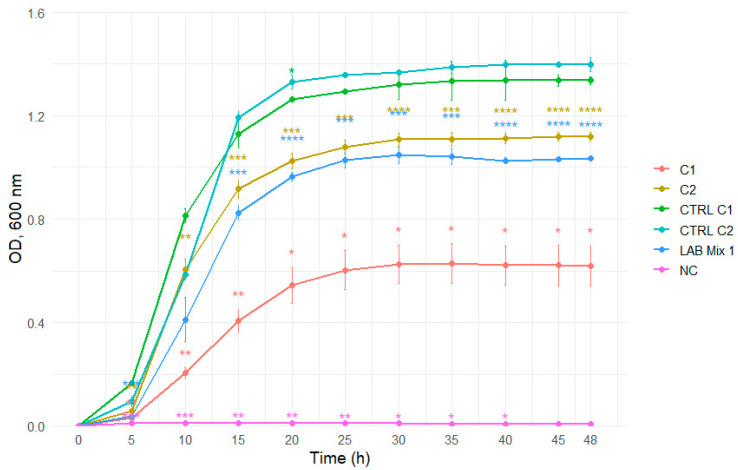
Growth curves of lactic acid bacteria strains C1, C2, and the mixed culture LAB Mix 1 in sucrose syrup at dilutions 1:2. Growth kinetics were recorded over 48 h and represent the mean of four biological replicates. Asterisks indicate statistical significance versus T0 (one-sample *t*-test, Bonferroni corrected). Significance levels are coded as follows: * *p* < 0.05; ** *p* < 0.01; *** *p* < 0.001; **** *p* < 0.0001.

**Figure 12:**
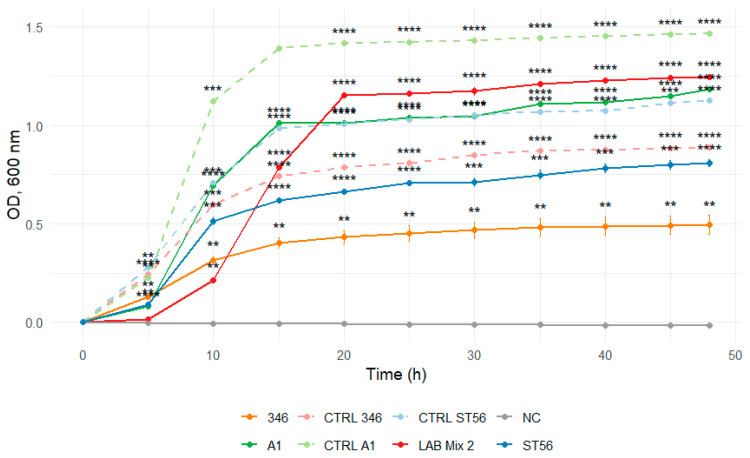
Growth curves of lactic acid bacteria strains A1, 346, ST56, and the mixed culture LAB Mix 2 in sucrose syrup at dilutions 1:2. Growth kinetics were recorded over 48 h and represent the mean of four biological replicates. Asterisks indicate statistical significance versus T0 (one-sample *t*-test, Bonferroni corrected). Significance levels are coded as follows: ** *p* < 0.01; *** *p* < 0.001; **** *p* < 0.0001.

In the most diluted condition (1:2 SS; Figure 11 and Figure 12), growth proceeded more rapidly, and both mixed cultures and several single strains reached levels comparable to the MRS controls, indicating that osmotic stress was largely alleviated at this sugar concentration.

**Figure 13:**
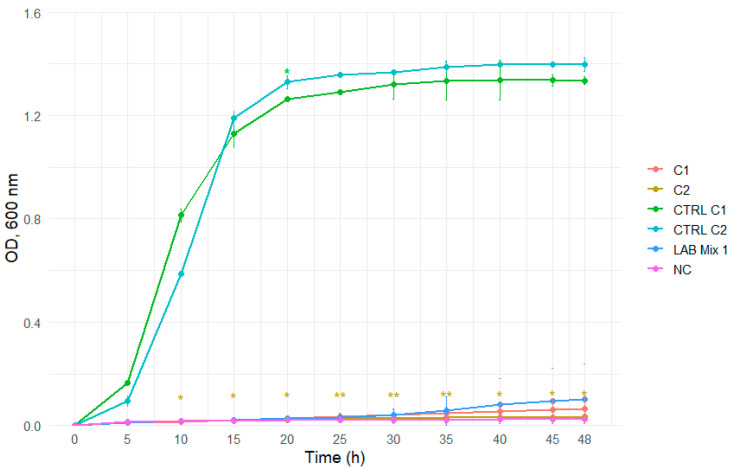
Growth curves of lactic acid bacteria strains C1, C2, and the mixed culture LAB Mix 1 in undiluted ApiSyrup. Growth kinetics were recorded over 48 h and represent the mean of four biological replicates. Asterisks indicate statistical significance versus T0 (one-sample *t*-test, Bonferroni corrected). Significance levels are coded as follows: * *p* < 0.05; ** *p* < 0.01.

**Figure 14:**
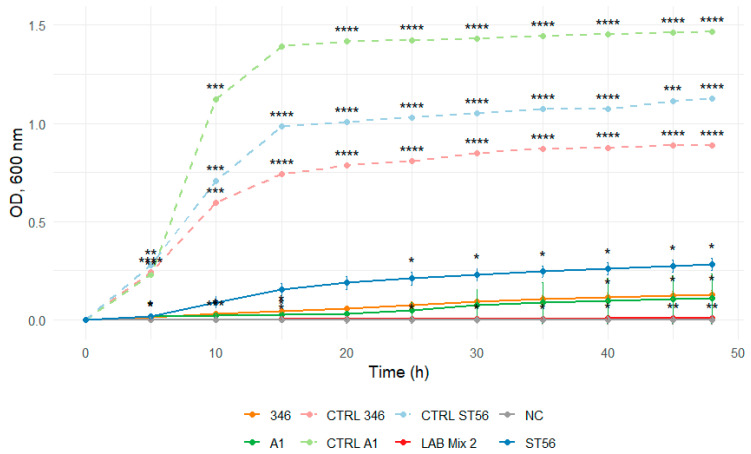
Growth curves of lactic acid bacteria strains A1, 346, ST56, and the mixed culture LAB Mix 2 in undiluted ApiSyrup. Growth kinetics were recorded over 48 h and represent the mean of four biological replicates. Asterisks indicate statistical significance versus T0 (one-sample *t*-test, Bonferroni corrected). Significance levels are coded as follows: * *p* < 0.05; ** *p* < 0.01; *** *p* < 0.001; **** *p* < 0.0001.

A similar pattern was observed for ApiSyrup. In undiluted AS (Figure 13 and Figure 14), growth was limited for all strains; however, mixed cultures consistently achieved higher OD values than single strains, suggesting superior tolerance.

**Figure 15:**
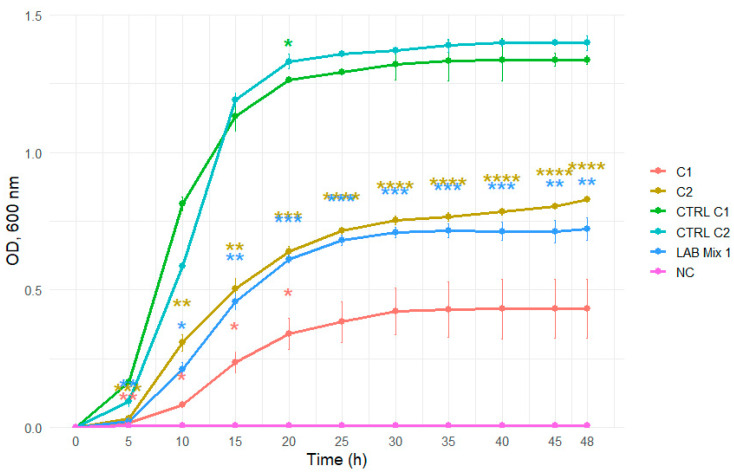
Growth curves of lactic acid bacteria strains C1, C2, and the mixed culture LAB Mix 1 in 1:1 diluted ApiSyrup. Growth kinetics were recorded over 48 h and represent the mean of four biological replicates. Asterisks indicate statistical significance versus T0 (one-sample *t*-test, Bonferroni corrected). Significance levels are coded as follows: * *p* < 0.05; ** *p* < 0.01; *** *p* < 0.001; **** *p* < 0.0001.

**Figure 16:**
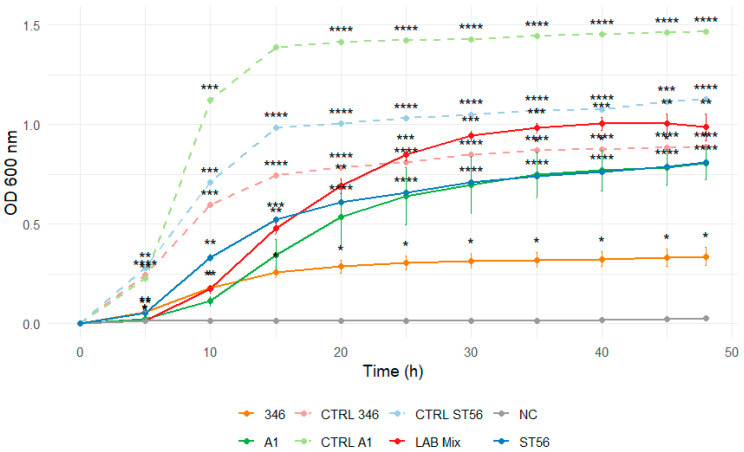
Growth curves of lactic acid bacteria strains A1, 346, ST56, and the mixed culture LAB Mix 2 in 1:1 diluted ApiSyrup. Growth kinetics were recorded over 48 h and represent the mean of four biological replicates. Asterisks indicate statistical significance versus T0 (one-sample *t*-test, Bonferroni corrected). Significance levels are coded as follows: * *p* < 0.05; ** *p* < 0.01; *** *p* < 0.001; **** *p* < 0.0001.

A 1:1 dilution markedly improved growth, again with mixed cultures outperforming individual strains (Figure 15 and Figure 16).

**Figure 18:**
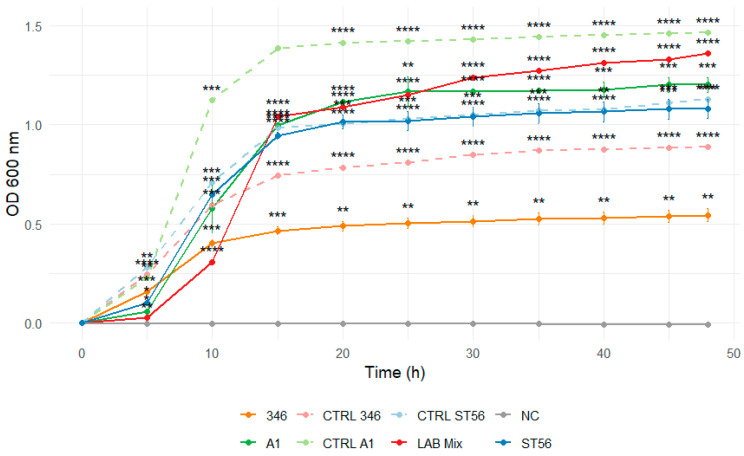
Growth curves of lactic acid bacteria strains A1, 346, ST56, and the mixed culture LAB Mix 2 in 1:2 diluted ApiSyrup. Growth kinetics were recorded over 48 h and represent the mean of four biological replicates. Asterisks indicate statistical significance versus T0 (one-sample *t*-test, Bonferroni corrected). Significance levels are coded as follows: * *p* < 0.05; ** *p* < 0.01; *** *p* < 0.001; **** *p* < 0.0001.

At the lowest concentration (1:2; Figure 17 and Figure 18), mixed cultures reached growth levels comparable to controls, while several single strains, particularly those exhibiting greater osmotolerance, also grew substantially.

Across both syrup types, mixed LAB cultures demonstrated enhanced growth under high-sugar, high-osmolarity conditions relative to single strains, and dilution of sugar matrices markedly improved performance for all LAB tested.

These results are consistent with established knowledge that high sugar concentrations generate hyperosmotic environments that reduce cellular water availability, disrupt homeostasis, and impede microbial proliferation [63,64,65]. Accordingly, the delayed growth and low maximal absorbance observed in 2:1 SS and undiluted AS are characteristic of strong osmotic stress. Prior studies examining LAB intended for apicultural use similarly reported survival in sugar syrups, often with strain-dependent variability and improved outcomes in diluted formulations [66]. Likewise, *Lp. plantarum* strains have been shown to tolerate sucrose concentrations up to 50% or glucose–fructose media at acidic pH, highlighting that some LAB possess intrinsic mechanisms for osmotic adaptation [34,67].

The superior performance of mixed cultures in our study suggests potential synergistic interactions that enhance robustness under osmotic stress. Such observations align with reports indicating that multi-strain LAB consortia do not display antagonism and may represent a promising approach advantageous for probiotic formulations in beekeeping [34,66]. Mechanistically, osmotic tolerance in LAB involves accumulation of compatible solutes, uptake of osmoprotectants, and metabolic adjustments supporting intracellular osmotic balance [63,64,68]. Although molecular pathways were not investigated here, the consistently higher growth of mixed cultures suggests complementary physiological strategies among strains, potentially resulting in improved collective resilience. Given that sugar syrups are widely used in apiculture for colony feeding, particularly during pre-winter periods or nutritional shortages, the viability of probiotic LAB in such matrices is a relevant practical consideration [62,66]. Our data indicate that undiluted syrups may severely limit LAB growth and thus compromise delivery efficiency. In contrast, moderate dilution (1:1 or 1:2) substantially improves growth performance, making these formulations more suitable for probiotic administration under controlled conditions. Furthermore, mixed cultures appear more robust across a range of sugar concentrations, offering greater flexibility for practical application without requiring extensive syrup adjustment by beekeepers.

Although the sugar syrup assays demonstrated sustained growth and metabolic activity of LAB under high osmotic pressure, these results are based on optical density measurements and should be interpreted as indicative of biomass increase rather than absolute viability. Enumeration of colony-forming units (CFU) would provide a more direct measure of survival and should be included in future studies. Moreover, while the observed tolerance to syrup conditions supports the feasibility of using such matrices as delivery vehicles, no conclusions regarding in vivo probiotic effectiveness can be drawn in the absence of validation under apicultural field conditions.

## 4. Conclusions

The results of this study indicate that the evaluated LAB strains possess a combination of safety, functional, and technological properties to support their consideration as probiotic candidates in a preliminary screening framework for potential use in beekeeping. These strains exhibited high auto-aggregation and surface hydrophobicity, particularly *A. kunkeei* and *F. fructosus*, traits commonly associated with adhesion-related properties in vitro, potential host interaction, and competitive interactions under experimental conditions. Mixed cultures showed intermediate-to-high values, suggesting functional compatibility and possible synergistic interactions under the tested conditions. No mutual antagonism was observed, indicating that these strains can coexist without inhibitory interference and might be compatible for multi-strain formulations. The organic acid profiles revealed strain-dependent metabolic specializations, with *Lp. plantarum* A1H1B2 and LAB mixtures producing the highest lactic and citric acid levels, metabolites previously associated in the literature with inhibitory activity against *P. larvae* and with modulation of *N. ceranae* infections, while *A. kunkeei* strains contributed substantial acetic acid production. Several LABs expressed peroxidase activity, suggesting a capacity to mitigate oxidative stress in the honey bee gut environment. All isolates were non-hemolytic, confirming a favourable safety profile consistent with probiotic criteria. Antibiotic susceptibility testing revealed predominantly intrinsic, non-transferable resistance patterns typical of LABs, though strain-level variability underscores the need for genomic screening prior to field application. Finally, growth assays in sugar-based feeding syrups showed that mixed cultures consistently outperformed single strains under hyperosmotic conditions and that moderate dilution of syrups markedly improved LABs’ viability, emphasizing the importance of formulation conditions for probiotic delivery. Overall, the results obtained highlight that the evaluated LAB, especially when combined, can be considered promising candidates for use in probiotic formulations to support the health and robustness of honey bees. However, translation into applied probiotic or bio-control strategies will require direct pathogen inhibition assays, comprehensive genomic safety evaluation, and field-based validation to ensure effectiveness.

## Figures and Tables

**Figure 17:**
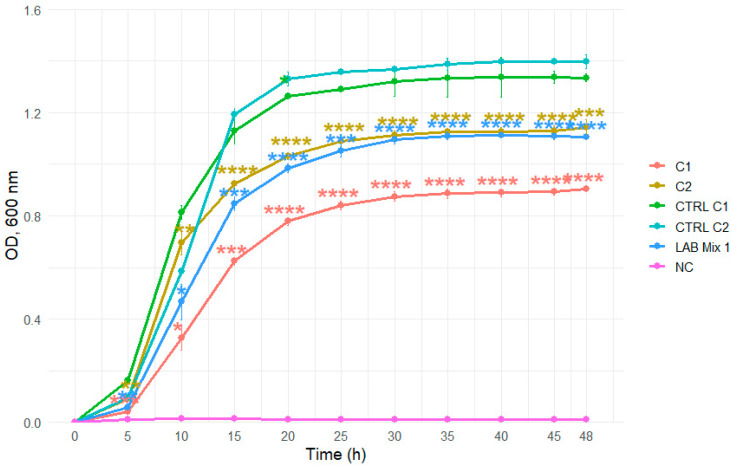
Growth curves of lactic acid bacteria strains C1, C2, and the mixed culture LAB Mix 1 in 1:2 diluted ApiSyrup. Growth kinetics were recorded over 48 h and represent the mean of four biological replicates. Asterisks indicate statistical significance versus T0 (one-sample *t*-test, Bonferroni corrected). Significance levels are coded as follows: * *p* < 0.05; ** *p* < 0.01; *** *p* < 0.001; **** *p* < 0.0001.

**Table 1:** Retention time, relative standard deviation in retention time (RSD %), R^2^, LOD, and LOQ of the organic acids.

Organic Acid	Retention Time (R_t_ min)	RSD% (R_t_ min)	R^2^	LOD (mg/L)	LOQ (mg/mL)
Lactic acid	7.8	0.12	0.9999	5.7	7.8
Acetic acid	8.1	0.17	0.9999	6.0	8.4
Citric acid	9.8	0.53	0.999	4.1	7.6

## Data Availability

The original contributions presented in this study are included in the article/Supplementary Material. Further inquiries can be directed to the corresponding authors.

## Supplementary Materials

The following supporting information can be downloaded at: https://www.mdpi.com/article/10.3390/microorganisms14030579/s1, Table S1: Auto-aggregation (AA) at 35 °C of the tested lactic acid bacteria (*A. kunkeei* C1, *Lactobacillus apis* C2, and LAB Mix 1). Data are expressed as mean ± SD (n = 3) and reported as %AA. Different lowercase letters within the same column and uppercase letters within the same row indicate significant differences (two-way ANOVA followed by Tukey’s post hoc test, *p* < 0.05); Table S2: Auto-aggregation (AA) at 35 °C of the tested lactic acid bacteria (*Lactiplantibacillus plantarum* A1H1B2, *Apilactobacillus kunkeei* ST56, *Fructobacillus fructosus* 346 and LAB Mix 2). The data (mean ± SD; n = 3) were expressed as percentage of AA, different lowercase letters in each column and uppercase letters in each row indicate significant differences (*p* < 0.05); Table S3: Hydrophobicity (%) of *Apilactobacillus kunkeei* C1, *Lactobacillus apis* C2, and LAB Mix 1 after different contact times with toluene and xylene. Data are expressed as mean ± SD (n = 3). Different lowercase letters within a row indicate significant differences among strains at the same time, and different uppercase letters within a column indicate significant differences among time points for the same strain (two-way ANOVA followed by Tukey’s post hoc test, *p* < 0.05); Table S4: Hydrophobicity (%) of *Lp. plantarum* A1H1B2, *A. kunkeei* ST56, *F. fructosus* 346, and LAB Mix 2 after different contact times with toluene and xylene. Data are expressed as mean ± SD (n = 3). Different lowercase letters within a row indicate significant differences among strains at the same time, and different uppercase letters within a column indicate significant differences among time points for the same strain (two-way ANOVA followed by Tukey’s post hoc test, *p* < 0.05); Table S5: PLC-PDA profile of organic acid production by lactic acid bacteria (LAB) strains. Lactic, acetic, and citric acids were quantified in the cell-free supernatant (CFS). Data are expressed as mean ± SD (n = 3). Different lowercase letters within each row indicate significant differences among strains for the same acid (one-way ANOVA followed by Tukey’s post hoc test, *p* < 0.05).
