# Supplementary material for: Probiotic Assessment of Lactic Acid Bacteria Strains and Consortia for Enhancing Honey Bee Health and Nutrition
Source: Microorganisms. 2026 Mar 4;14(3):579. doi: 10.3390/microorganisms14030579 (PMC13028829; doi:10.3390/microorganisms14030579)
Supplement: Supplementary file 1 [file microorganisms-14-00579-s001.zip › Table S1.pdf]

microorganisms MDPI

Table S1. Auto-aggregation (AA) at 35 °C of the tested lactic acid bacteria (*A. kunkeei* C1, *Lactobacillus apis* C2, and LAB Mix 1). Data are expressed as mean ± SD (n = 3) and reported as %AA. Different lowercase letters within the same column and uppercase letters within the same row indicate significant differences (two-way ANOVA followed by Tukey's post hoc test, $p < 0.05$).

	Bacterial strains		
Time (h)	*A. kunkeei* C1	*L. apis* C2	LABs Mix 1
1	8.7 ± 0.3^{Ad}	6.3 ± 0.9^{Bd}	4.0 ± 0.5^{Cd}
2	10.3 ± 0.3^{Bc}	12.2 ± 0.9^{Bc}	20.6 ± 1.0^{Ac}
5	22.0 ± 0.5^{Bb}	23.4 ± 0.9^{Bb}	39.6 ± 0.8^{Ab}
24	57.9 ± 0.3^{Aa}	49.3 ± 0.5^{Ca}	54.0 ± 0.2^{Ba}
