# Supplementary material for: Probiotic Assessment of Lactic Acid Bacteria Strains and Consortia for Enhancing Honey Bee Health and Nutrition
Source: Microorganisms. 2026 Mar 4;14(3):579. doi: 10.3390/microorganisms14030579 (PMC13028829; doi:10.3390/microorganisms14030579)
Supplement: Supplementary file 1 [file microorganisms-14-00579-s001.zip › Table S2.pdf]

microorganisms

Table S2. Auto-aggregation (AA) at 35 °C of the tested lactic acid bacteria (*Lactiplantibacillus plantarum* A1H1B2, *Apilactobacillus kunkeei* ST56, *Fructobacillus fructosus* 346 and LAB Mix 2). The data (mean ± SD; $n = 3$) were expressed as percentage of AA, different lowercase letters in each column and uppercase letters in each row indicate significant differences ($p < 0.05$).

	Bacterial strains			
Time (h)	*Lp. plantarum* A1H1B2	*A. kunkeei* ST56	*F. fructosus* 346	LAB Mix 2
1	2.2 ± 0.2^{Dd}	5.8 ± 0.2^{Bd}	10.2 ± 0.3^{Ad}	4.6 ± 0.2^{Cd}
2	7.0 ± 0.6^{Dc}	16.9 ± 0.3^{Bc}	22.1 ± 0.1^{Ac}	12.0 ± 0.2^{Cc}
5	11.8 ± 0.8^{Db}	26.8 ± 0.9^{Bb}	42.7 ± 0.9^{Ab}	22.7 ± 0.9^{Cb}
24	30.0 ± 0.8^{Da}	60.7 ± 0.4^{Ca}	75.6 ± 0.3^{Aa}	65.8 ± 0.3^{Ba}
