# Supplementary material for: Probiotic Assessment of Lactic Acid Bacteria Strains and Consortia for Enhancing Honey Bee Health and Nutrition
Source: Microorganisms. 2026 Mar 4;14(3):579. doi: 10.3390/microorganisms14030579 (PMC13028829; doi:10.3390/microorganisms14030579)
Supplement: Supplementary file 1 [file microorganisms-14-00579-s001.zip › Table S3.pdf]

microorganisms

MDPI

Table S3. Hydrophobicity (%) of *Apilactobacillus kunkeei* C1, *Lactobacillus apis* C2, and LAB Mix 1 after different contact times with toluene and xylene. Data are expressed as mean ± SD (n = 3). Different lowercase letters within a row indicate significant differences among strains at the same time, and different uppercase letters within a column indicate significant differences among time points for the same strain (two-way ANOVA followed by Tukey's post hoc test, $p < 0.05$).

	**Hydrophobicity (%) Toluen**		
Time (min)	*Apilactobacillus kunkeei* C1	*Lactobacillus apis* C2	LABs Mix 1
15	32.9 ± 0.6^{Cc}	70.3 ± 0.1^{Bc}	74.8 ± 0.2^{Ac}
30	48.3 ± 0.5^{Cb}	84.2 ± 0.1^{Bb}	88.6 ± 0.2^{Ab}
60	58.9 ± 0.5^{Ca}	91.8 ± 0.5^{Ba}	94.5 ± 0.2^{Aa}

	**Hydrophobicity (%) (Xylen)**		
Time (min)	*Apilactobacillus kunkeei* C1	*Lactobacillus apis* C2	LAB Mix 1
15	26.0 ± 0.5^{Cc}	63.7 ± 0.4^{Bc}	69.9 ± 0.2^{Ac}
30	41.0 ± 0.3^{Cb}	79.1 ± 0.3^{Bb}	84.8 ± 0.1^{Ab}
60	50.1 ± 0.3^{Ca}	88.2 ± 0.2^{Ba}	92.0 ±0.2^{Aa}
