# Supplementary material for: Probiotic Assessment of Lactic Acid Bacteria Strains and Consortia for Enhancing Honey Bee Health and Nutrition
Source: Microorganisms. 2026 Mar 4;14(3):579. doi: 10.3390/microorganisms14030579 (PMC13028829; doi:10.3390/microorganisms14030579)
Supplement: Supplementary file 1 [file microorganisms-14-00579-s001.zip › Table S4.pdf]

microorganisms

MDPI

Table S4. Hydrophobicity (%) of *Lp. plantarum* A1H1B2, *A. kunkeei* ST56, *F. fructosus* 346, and LAB Mix 2 after different contact times with toluene and xylene. Data are expressed as mean ± SD (n = 3). Different lowercase letters within a row indicate significant differences among strains at the same time, and different uppercase letters within a column indicate significant differences among time points for the same strain (two-way ANOVA followed by Tukey's post hoc test, $p < 0.05$).

	**Hydrophobicity (%) Toluen**			
Time (min)	*Lp. plantarum* A1H1B2	*A. kunkeei* ST56	*F. fructosus* 346	LAB Mix 2
15	57.1 ± 0.7^{Bc}	90.2 ± 0.4^{Ac}	28.3 ± 0.5^{Dc}	32.7 ± 0.8^{Cc}
30	64.0 ± 0.8^{Bb}	96.2 ± 0.2^{Ab}	45.6 ± 0.8^{Db}	56.4 ± 0.8^{Cb}
60	66.8 ± 0.3^{Da}	97.1 ± 0.2^{Aa}	70.8 ± 0.3^{Ca}	82.3 ± 0.3^{Ba}

	**Hydrophobicity (%) Xylen**			
Time (min)	*Lp. plantarum* A1H1B2	*A. kunkeei* ST56	*F. fructosus* 346	LAB Mix 2
15	69.3 ± 0.5^{Bb}	96.7 ± 0.4^{Ab}	37.8 ± 0.3^{Dc}	41.0 ± 0.6^{Cc}
30	75.1 ± 0.3^{Ba}	98.5 ± 0.4^{Aa}	57.3 ± 0.3^{Db}	70.5 ± 0.4^{Cb}
60	75.6 ± 0.3^{Da}	98.3 ± 0.3^{Ba}	80.8 ± 0.3^{Ca}	99.1 ± 0.3^{Aa}
