# Supplementary material for: Probiotic Assessment of Lactic Acid Bacteria Strains and Consortia for Enhancing Honey Bee Health and Nutrition
Source: Microorganisms. 2026 Mar 4;14(3):579. doi: 10.3390/microorganisms14030579 (PMC13028829; doi:10.3390/microorganisms14030579)
Supplement: Supplementary file 1 [file microorganisms-14-00579-s001.zip › Table S5.pdf]

microorganisms

Table S5. PLC-PDA profile of organic acid production by lactic acid bacteria (LAB) strains. Lactic, acetic, and citric acids were quantified in the cell-free supernatant (CFS). Data are expressed as mean ± SD (n = 3). Different lowercase letters within each row indicate significant differences among strains for the same acid (one-way ANOVA followed by Tukey's post hoc test, $p < 0.05$).

Organic acids	Producer strain	Concentration (g/L)
Lactic acid	*A. kunkeei* C1	16.98 ± 0.18[e]
	L. apis C2	23.61 ± 0.15[b]
	LAB Mix 1	19.80 ± 0.20[c]
	Lp. plantarum A1H1B2	25.46 ± 0.34[a]
	A. kunkeei ST56	18.45 ± 0.23[d]
	F. fructosus 346	17.36 ± 0.17[e]
	LAB Mix 2	19.80 ± 0.24[c]
Citric acid	*A. kunkeei* C1	1.46 ± 0.01[b]
	L. apis C2	1.41 ± 0.01[c]
	LAB Mix 1	1.58 ± 0.02[a]
	Lp. plantarum A1H1B2	1.57 ± 0.03[a]
	A. kunkeei ST56	1.37 ± 0.02[c]
	F. fructosus 346	1.32 ± 0.01[d]
	LAB Mix 2	1.47 ± 0.02[b]
Acetic acid	*A. kunkeei* C1	0.44 ± 0.01[a]
	L. apis C2	0.08 ± 0.00[g]
	LAB Mix 1	0.39 ± 0.01[b]
	Lp. plantarum A1H1B2	0.23 ± 0.00[f]
	A. kunkeei ST56	0.26 ± 0.01[e]
	F. fructosus 346	0.35 ± 0.00[c]
	LAB Mix 2	0.32 ± 0.01[d]

 https://doi.org/10.3390/microorganisms14030579
